# Video head impulse test in bilateral vestibulopathy

**DOI:** 10.1016/j.bjorl.2020.05.014

**Published:** 2020-06-18

**Authors:** Mayada Elsherif, Mirhan Eldeeb

**Affiliations:** Alexandria University, Audiovestibular Unit, Department of Otorhinolaryngology, Egypt

**Keywords:** Vestibulo-ocular reflex, Saccadic eye movements, Bilateral vestibulopathy

## Abstract

**Introduction:**

Bilateral vestibulopathy is a rare chronic condition with multiple etiologies. Bilateral vestibulopathy is characterized mainly by unsteadiness when walking or standing, which worsens in darkness, as well as oscillopsia. The degree of handicap caused by bilateral vestibulopathy is variable and remains controversial.

**Objectives:**

To determine the value of the video Head Impulse Test in quantifying vestibular deficit and to establish its impact on the quality of life.

**Methods:**

Twenty patients (mean age, 41.9 years; range 14–80 years) fulfilling the recent Barany criteria of bilateral vestibulopathy, responded to the Situational Vertigo Questionnaire and underwent vestibular examination including fixation, positional tests, oculomotor test battery and video head impulse test.

**Results:**

The relation between each of the video head impulse test parameters and the scores from the questionnaire were statistically analyzed. We observed that patients with covert saccades on the video head impulse test were more likely to have a better quality of life than those with both covert and overt saccades, regardless of the vestibulo-ocular reflex gain in each semicircular canal. The presence of covert saccades was found to be associated with an improved quality of life regardless of the severity of vestibule ocular reflex-deficit. Our conclusion was that vestibule ocular reflex gain, measured by video head impulse test, does not quantify the severity of affection of quality of life in patients with bilateral vestibulopathy.

**Conclusion:**

Covert saccades are strategies aiming at minimizing the blurring of vision during head movement, that is an adaptive mechanism that improves quality of life. Therefore, we recommend that video head impulse test should be a part of the routine diagnostic workup of bilateral vestibulopathy.

## Introduction

Bilateral Vestibulopathy (BVP) is a rare, heterogeneous, chronic vestibular disorder with different etiologies.^1^, ^2^ Many synonyms, such as bilateral vestibular weakness, bilateral vestibular hypofunction, and bilateral vestibular loss, have been used to describe the condition. A recent consensus statement from the Barany society refers to the condition as bilateral vestibulopathy.^3^ BVP is caused by partial or complete deficit of both vestibular organs or vestibular nerves, or a combination of both.^3^ Patients with BPV experience variable symptoms, such as oscillopsia, unsteadiness while walking, darkness evoked disequilibrium, and attacks of vertigo.^4^ BVP poses a significant diagnostic challenge.^5^ It is frequently misdiagnosed in outpatient clinics due to its diverse clinical characteristics and vague symptomatology.^6^ Although different labyrinthine components are involved, there are no definitive quantitative criteria for diagnosing BVP.^2^ Previous studies have preferred the Video Head Impulse Test (vHIT) over other Vestibulo-Ocular Reflex (VOR) assessment tools in establishing the diagnosis of both unilateral vestibular hypofunction and BVP.^7^, ^8^ However, one recent study recommends the use of a combination of various vestibular tests to reveal the site of the lesion in BVP.^9^

The degree of handicap caused by BVP and its impact on a patient’s Quality Of Life (QOL) remain controversial.^10^ Patients with BVP usually have an increased risk of falling, which makes routine activities difficult.^11^ Gillespie and Minor reported that prediction of BVP prognosis is multifactorial, depending heavily on the course of the disorder and the severity of the lesion.^12^

The purpose of the present study was to explore the association between vHIT results and the degree of handicap caused by BVP. We hypothesized that vHIT could be used to quantify the severity of BVP and its impact on QOL. If this relationship exists, Vhit could be a useful preliminary test to quantify the severity of BVP, which is important for patient rehabilitation.

## Methods

This was a cross-sectional observational study aimed to explore the association between the quantity of VOR deficit measured via vHIT, and the impact of BVP on QOL.

### Subjects

This study was performed in the audio-vestibular unit of the Department of Otorhinolaryngology at the Main University Hospital in Alexandria, Egypt. The study was approved by the Ethics Committee of the Faculty of Medicine at Alexandria University in Egypt, IRB number 00007555. Twenty patients, fulfilling the recent Barany criteria of BVP, were included in this study. The enrolled patients presented with a minimum of 3 months of clinical history symptoms. All patients responded to the SVQ and pure tone audiometry. All subjects were evaluated using Videonystagmography (VNG) and vHIT.

### Methods

Scoring of the dizziness complaint was performed using a translated form of the SVQ.^13^ The SVQ is a 19 item questionnaire aiming specifically at recognizing visual vertigo, in which patients present with increased visual dependence because of poor vestibular compensation.^14^ The SVQ was chosen for this study because it indicates the degree to which different situations affect patient’s symptoms negatively.

The impact on the patient’s QOL was classified as moderate if the symptoms mildly affected the patient’s routine daily activities, and as severe, if the patient needed assistance while performing those routine activities. Pure tone threshold sensitivity was measured. VNG examination was performed using the ICS Impulse 3-Dimensional vHIT system (GN Otometrics, Taastrup, Denmark). The normal VOR-gain was > 0.8 for the lateral and > 0.7 for the vertical canals, as specified by the manufacturer.

Fixation testing in neutral eye position, with and without vision, and positioning testing were performed. The battery of oculomotor tests (saccade, tracking, Optokinetic nystagmus [OKN], and gaze-evoked nystagmus via a red LED light bar) and vHIT using ICS Impulse (GN Otometrics, Taastrup, Denmark) were performed. Regarding vHIT, both lateral and vertical canal stimulation tests were performed via passive head thrusts at 10°‒20° and a velocity range of 150°‒200°/sec.

### Statistical analysis

The Chi-Square test was used to study the relationship between the type of saccade and its impact on QOL. The *t*-test was used to study both the relationship between the VOR-gain in each canal and its accompanying impact on QOL, as well as the relationship between the type of saccade and the associated SVQ score.

## Results

### Clinical history analysis

Twenty patients (12 males [60%] and 8 females [40%], mean age 41.9 years and age range 14‒80 years) were included in this study. Etiology of BVP for each of the 20 patients is shown in Table 1.

**Table 1:** Patient’s age, sex, canals affected, etiology, type of saccades, score on the questionnaire, and the impact on Quality Of Life (QOL).

Sex	Age	Canals with VOR loss	Etiology of BVP	Nystagmus on fixation and positional test	Type of saccades	Score on questionnaire	Impact on QOL
F	20	All six canals	Usher syndrome	No nystagmus	Both	72	Severe
M	14	All six canals	Usher syndrome	No nystagmus	Covert	56	Moderate
F	45	Spared anterior canal	Idiopathic	Nystagmus	Covert	56	Moderate
M	34	All six canals	Ototoxicity	No nystagmus	Both	67	Severe
M	35	All six canals	Idiopathic	No nystagmus	Covert	50	Moderate
M	70	All six canals	Idiopathic	No nystagmus	Both	70	Severe
M	50	All six canals	Idiopathic	No nystagmus	Covert	58	Moderate
F	58	All six canals	Idiopathic	No nystagmus	Both	70	Severe
F	50	All six canals	Idiopathic	No nystagmus	Both	72	Severe
F	40	All six canals	Neurofibromatosis type 2	No nystagmus	Both	70	Severe
M	45	All six canals	Idiopathic	No nystagmus	Covert	56	Moderate
M	30	Spared anterior canal	Idiopathic	Nystagmus	Covert	50	Moderate
M	24	Spared anterior canal	Idiopathic	Nystagmus	Covert	56	Moderate
M	22	Spared anterior canal	Idiopathic	Nystagmus	Both	69	Moderate
M	80	Spared anterior canal	Idiopathic	Nystagmus	Both	72	Severe
M	40	All six canals	Ototoxicity	No nystagmus	Covert	50	Moderate
F	55	Spared anterior canal	Idiopathic	Nystagmus	Covert	56	Moderate
F	36	All six canals	Ototoxicity	No nystagmus	Both	70	Severe
M	50	All six canals	Idiopathic	No nystagmus	Covert	56	Moderate
F	40	All six canals	Idiopathic	No nystagmus	Covert	50	Moderate

VOR, Vestibulo-Ocular Reflex; QOL, Quality of Life; M, Male; F, Female.

### Pure tone audiogram

Six subjects showed bilateral normal hearing thresholds and one subject with bilateral schwannomas showed asymmetrical, moderately severe Sensorineural Hearing Loss (SNHL). The two subjects with Usher syndrome had unilateral cochlear implants with the other ear being profoundly deaf. Eight subjects had different levels of hearing loss, varying from mild to moderately severe SNHL.

### Video head impulse test analysis

#### Vestibulo-ocular reflex gain

Fourteen patients (70%) showed a low VOR-gain in all six canals. The remaining six patients (30%) with idiopathic etiology showed no affection of the anterior canals on vHIT.

#### Catch-up saccades

Three types of saccades were reported: during vHIT (covert), after vHIT (overt), and a combination of during and after vHIT (scattered covert and overt saccades).

#### Videonystagmography

None of the patients showed spontaneous nystagmus with vision. Six patients (30%) showed spontaneous nystagmus with vision denied. Patients with unilaterally spared anterior canal showed torsional downbeat nystagmus toward the spared canal.

Table 1 lists the patients’ age, sex, etiology of BVP, affected canals, type of saccades, SVQ score, and QOL impact. All but four patients showed normal oculomotor test battery results ‒ the remaining 4 (20%) showed low gain symmetrical pursuit.

### Statistical analysis

The *t*-test performed to assess the relationship between type of saccades and SVQ score revealed a predictable relationship between the presence of covert saccades and a higher SVQ score (Table 2). The *t*-test performed to assess the relationship between the mean VOR-gain in each semicircular canal and QOL impact revealed no statistically significant relationship, except between the right lateral canal VOR-gain and QOL impact (Table 3).

**Table 2:** Relation between type of saccades and SVQ score questionnaire.

Score questionnaire	Type of saccades		*t*	*p*
	Both overt and covert saccades (n = 9)	Covert saccades (n = 10)		
Median (Min–Max)	70 (67–72)	56 (50–58)	14.540	<0.001^a^
Mean±SD	70.2±1.6	54±3.2		

*t*, Student *t*-test; p, p-value for comparing between the two categories.

a Statistically significant at *p* ≤  0.05.

**Table 3:** The relation between different vHIT parameters and the impact on quality of life (n = 20).

	Impact on QOL		Test of sig.	*p*
	Severe (n = 8)	Moderate (n = 12)		
Left lateral canal				
Median (Min–Max)	0.5 (0.3–0.6)	0.3 (0.1–0.7)	*t*=1.668	0.113
Mean±SD	0.5±0.1	0.3±0.2		
Right lateral canalL				
Median (Min–Max)	0.5 (0.2–0.6)	0.2 (0.1–0.6)	*t* = 2.544^a^	0.020^a^
Mean±SD	0.5±0.2	0.3±0.2		
Left posterior canal				
Median (Min–Max)	0.5 (0.1–0.6)	0.3 (0.1–0.5)	*t*=1.178	0.254
Mean±SD	0.4±0.2	0.3±0.2		
Right posterior canal				
Median (Min–Max)	0.4 (0.04–0.7)	0.2 (0.03–0.6)	*t*=0.785	0.442
Mean±SD	0.4±0.2	0.3±0.2		
Left anterior canal				
Median (Min–Max)	0.5 (0.1–0.8)	0.5 (0.2–1)	*t*=1.070	0.299
Mean SD	0.4±0.2	0.6±0.3		
Right anterior canal				
Median (Min–Max)	0.4 (0.2–0.7)	0.4 (0.1–1)	*t*=0.415	0.683
Mean±SD	0.4±0.2	0.5±0.3		

*t*, Student *t*-test; p, p-value for comparing between the two categories.

a Statistically significant at *p* ≤  0.05.

The Chi-Square test revealed a significant relationship between patients with only covert saccades and lower QOL impact (Table 4).

**Table 4:** Chi-Square test, representing the relation between the type of saccades and the impact on QOL.

	Impact on QOL		Test of sig.	*p*
	Severe (n = 8)	Moderate (n = 12)		
Type of saccades				
Both	8 (100%)	1 (8.3%)	χ^2^ = 16.296^a^	<0.001^a^
Covert	0 (0%)	11 (91.7%)		

χ^2^, Chi-square test; p, p-value for comparing between the two categories.

a Statistically significant at *p* ≤ 0.05.

The proportion of BVP patients whose QOL was severely impacted with both covert and overt saccades was 100% (n = 8), whereas the proportion of BVP patients whose QOL was severely impacted with only covert saccades was 0%. The difference in proportions was statistically significant (χ² [1, n = 20] = 16.29, *p* < 0.001).

## Discussion

Balance problems often profoundly affect a person’s daily activities.^15^ Patients with abnormal balance have a fear of falling and thus avoid multiple types of situations and activities.^16^

Sun et al. studied QOL in patients with BVP using the dizziness handicap inventory and established significant impact on QOL due to the increased risk of fall.^11^ In this study, we chose the SVQ, which was designed for the recognition of visual vestibular mismatch.

As patients with BVP show increased visual dependence, compensatory saccades generated after bilateral vestibular deficits are much more dependent on vision.^17^ Thus, patients with BVP show visual vestibular mismatch. In this study, we chose the SVQ because it was designed for recognizing similar visual vestibular mismatch.

Although aging is known to affect the quality of life, this effect may wane by controlling other factors like longstanding illness and social context.^18^ In this study, we studied a heterogenous group of patients with BVP who had variable age and etiology. Our subjects included two elderly patients (70 and 80 years old) with no history of longstanding disease or motor limitation. Another two patients (14 and 20 years old) had Usher syndrome, in whom the visual affection was very mild given that they only complained of difficulty with night vision. Retinitis pigmentosa, caused by Usher syndrome, is known to initially present with difficulty seeing in low light (“night blindness”).^19^

The gold standard for diagnosing Bilateral Vestibular Hypofunction (BVH) and defining its severity is the rotary chair testing. Computerized Dynamic Posturography has been used to assess overall balance in BVP patients. A study by Sprenger et al. found a direct proportional relationship between the postural sway and the vestibular impairment, when recording the center of sway in BVP patients using posturography.^20^

Caloric test, vHIT, and dynamic visual field test are adjuvant tests in diagnosing BVH.^5^ However, the caloric test has no role either in assessing the severity or in predicting the prognosis of BVP as vestibular compensation occurs mainly in daily activities (high frequency stimuli), which may modulate the vHIT results not the caloric.^21^

Using vHIT is advantageous for testing each of the semicircular canals separately. Also, aging has no effect on the VOR measured by vHIT, except for faster head impulses in subjects older than 70 years.^22^ Additionally, the appearance of refixation saccades was reported in elderly patients as a sign of semicircular canal dysfunction.^22^, ^23^ The refixation saccades in elderly patients have been explained by the lack of saccade suppression.^23^

Weber et al. studied the relationship between vHIT gain and caloric responses in cases of gentamicin vestibulotoxicity and found an agreement between both tests.^7^ In addition, a study performed by Moon et al. found an association between vHIT gain and caloric responses in the studied groups, with increased improvement from bilateral to unilateral to normal caloric responses.^24^ The variable rate of involvement of the semicircular canals, sparing the anterior canals in BVP, suggests the importance of vHIT in diagnosing and identifying BVP etiology.^25^

However, in the present study, the vHIT gains were not a reflection of the severity of BVP, inconsistent with the results of other studies. A study on 20 patients with BVH showed an association between low vHIT gains and the severity of BVP.^26^

The impact of covert saccades on visual performance in patients with BVP has been studied using dynamic visual acuity and vHIT.^27^ One study highlighted the significant role of covert saccades in improving the dynamic visual acuity in these patients.^27^

Additionally, covert saccades have been shown to decrease 37% of gaze error in patients with vestibular deficits.^28^

The present study demonstrated that the presence of covert saccades was associated with a moderate QOL impact and a lower SVQ score. Conversely, the presence of overt saccades was mostly associated with a severe QOL impact. Covert saccades, thus, play an important role in improving BVP symptomatology. This is in agreement with another study by Herman et al. that demonstrated the value of covert saccades in improving the dynamic visual acuity in BVP and in compensating for vestibular deficits.^27^ The modification of saccades after the vestibular loss has been shown to improve motor adaptation.^29^ The cerebellar visual error feedback loop is involved in modulating the latency of saccades.^30^, ^31^, ^32^ Covert saccades are strategies to minimize blurring of vision during head movement, making them an adaptive mechanism improving a patient’s QOL.

Thus, symptoms of BVP and their QOL impact should improve with time, regardless of the severity of the vestibular deficit. VOR-gain, as measured by vHIT, does not quantitatively reflect the severity of BVP. Future studies, involving larger numbers of BVP patients, are needed to validate our findings.

## Conclusions

The type of saccades measured by vHIT could reflect the severity of BVP and its impact on QOL. We recommend including vHIT in the diagnosis of BVP, as it is currently the only test able to evaluate the involvement of each semicircular canal and sparing of the vertical canals.

### Conflicts of interest

The authors declare no conflicts of interest.
